# A Laboratory Module for Physical Chemistry and Analytical Chemistry: The Kinetics of Aspirin Hydrolysis and Its Quantitation in Pharmaceutical Tablets

**DOI:** 10.1021/acs.jchemed.4c00809

**Published:** 2025-01-31

**Authors:** Victoire Delattre, Remi Olivier Labeille, Nicholas Slade Shropshire, Kyra Grace Kaiser, Brent Kirkland, Keith Zvoch, Ioana Emilia Pavel

**Affiliations:** † Department of Physical and Environmental Sciences, College of Science, Texas A&M University at Corpus Christi, 6300 Ocean Drive, Corpus Christi, Texas 78412-5800, United States; ‡ Department of Education Studies, College of Education, University of Oregon, 1215 University of Oregon, Eugene, Oregon 97403-1215, United States

**Keywords:** Upper-Division Undergraduate, Laboratory Instruction, Interdisciplinary/Multidisciplinary, Hands-On Learning/Manipulatives, Physical Chemistry, Analytical Chemistry

## Abstract

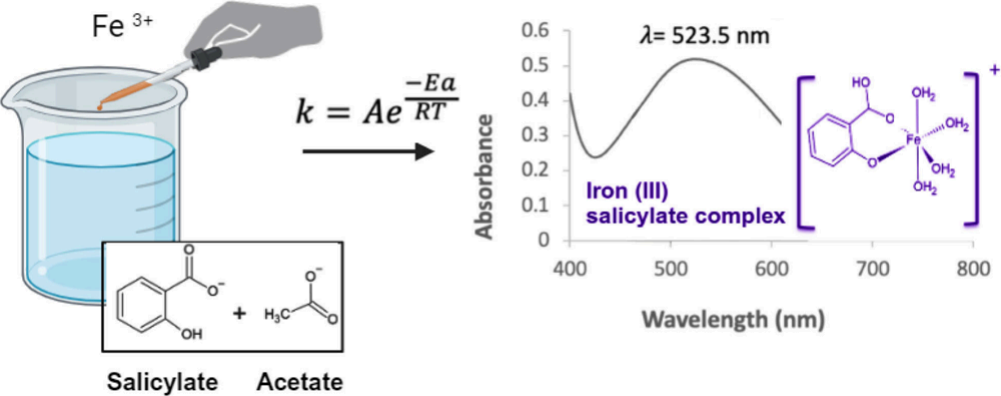

In this three-component laboratory module, upper-division chemistry students were introduced to the kinetics of the aspirin hydrolysis reaction and determined the concentration of its active pharmaceutical ingredient (acetylsalicylic acid-ASA) using a modern, benchtop ultraviolet–visible (UV–vis) absorption spectrophotometer. In the first component, students prepared analyte solutions from over-the-counter aspirin tablets and a relevant number of standards (n = 9–10) through both serial and parallel dilutions. In the second component, the ASA concentrations of three over-the-counter formulations (325 mg per tablet) were determined with percent differences as small as 1.1% using the Beer–Lambert law and external calibration curves. In the third component, students evaluated the reaction order (pseudo-first order), the rate constant (e.g., k *=* 3.0 × 10^–4^ s^–1^ at 75 °C), and the activation energy (*E*_a_ ∼ 67.3 kJ mol^–1^) of the hydrolysis reaction of ASA at various temperatures (e.g., 25, 37, 50, 75, and 85 °C). The last component was completed using a student-centered instructional approach, namely, process-oriented guided-inquiry learning (POGIL), which helped refine students’ research process skills and both basic and in-depth laboratory skills (weighing, solution handling, micropipetting, operation of a pH meter and a modern, benchtop absorption spectrophotometer). The student and instructor evaluations indicated a positive learning experience and high interest in this laboratory that was inspired by the quality control and quality assurance of pharmaceutical drugs.

## Introduction

Traditionally, the concepts presented in lectures are further explored and applied within the context of instructor-led, laboratories.^1−4^ These laboratory experiences provide students with both cognitive abilities and essential technical skills for subsequent laboratory courses, future graduate studies, and professional employment. The American Chemical Society (ACS) deems laboratory skills critical and emphasizes the importance of a robust laboratory experience across all areas of chemistry.^3,4^ In fact, a minimum of 350 h of hands-on laboratory experiences beyond general chemistry are required for the ACS-certified bachelors’ degrees. The upper-division, undergraduate laboratory module presented herein addresses this need by fine-tuning hands-on, key laboratory skills and offering in person training on sizable, modern instrumentation that is commonly employed by various science research and industry laboratories worldwide.^5−7^ This laboratory experiment was developed and successfully implemented in a *Physical Chemistry I Laboratory* course for two groups of students (from different academic years), but its three components (∼2–3 h each) can be individually or jointly adapted for other upper division, undergraduate laboratory courses. The overall goal was to introduce students to the kinetics of the hydrolysis reaction of aspirin tablets (*Physical Chemistry* laboratory) and to determine the concentration of its active pharmaceutical ingredient (*Analytical Chemistry, Pharmaceutical Chemistry, Biochemistry,* and *Forensic Chemistry* laboratories) using a modern, benchtop ultraviolet–visible (UV–vis) absorption spectrophotometer. Within this context, assessments were administered to evaluate the overall learning experience, the theoretical knowledge, the research process, and a set of predetermined basic and in-depth laboratory skills acquired by the students. In the first component, students were requested to prepare aspirin solutions and a set of standards through both serial and parallel dilutions of small volumes of analyte. In the second component, the concentration of the active pharmaceutical ingredient in over-the-counter aspirin formulations was estimated via interpolation from calibration curves and with the help of the Beer–Lambert law. In the third component, the kinetics of the hydrolysis reaction of aspirin was studied at different temperatures (*i.e*., 25, 37, 50, 75, and 85 °C) to establish the overall reaction order, the rate constant (k), and the activation energy (*E*_a_).

**Figure 1:**
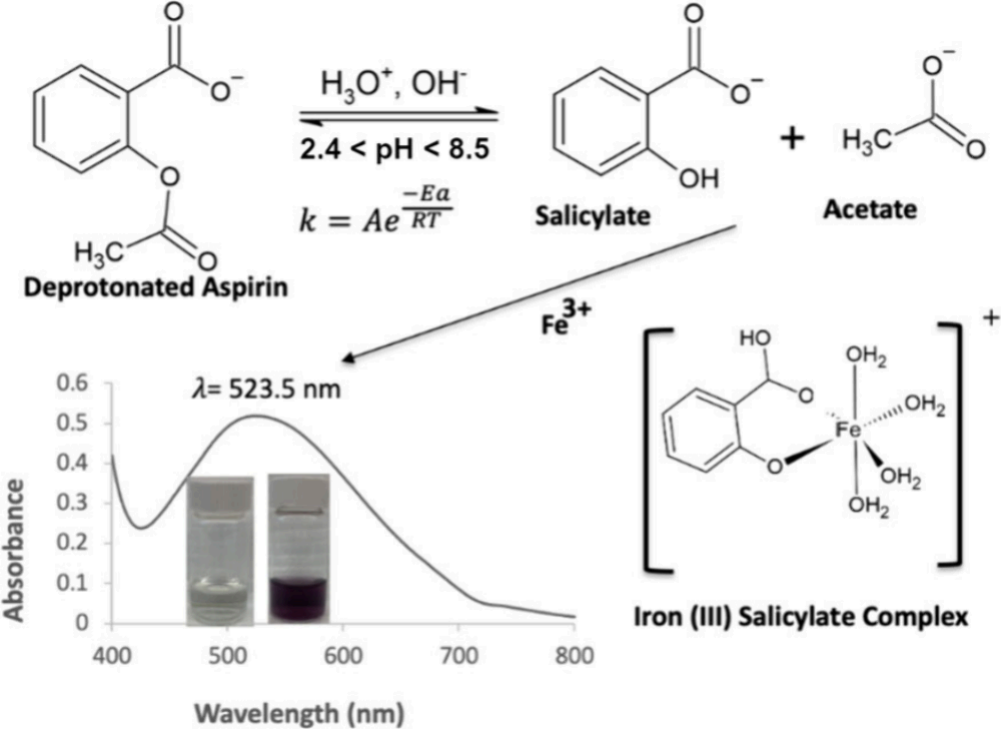
Schematic for the formation reaction of an iron(III) salicylate complex, which is spectrophotometrically active in the VIS region (λ_max_ = 523.5 nm). The reaction sequence shows the hydrolysis of acetylsalicylic acid (ASA) to salicylate (SA) and acetate ions, followed by the binding of the SA ion to Fe^3+^ to form the iron(III) salicylate complex. Inset shows two vials containing a colorless solution (0.160 mM, left) before the addition of iron(III) nitrate and a purple aqueous standard of iron(III) salicylate complex (0.160 mM, right). The aqueous ASA hydrolysis can be catalyzed by hydronium ions or hydroxide ions.^15^

Aspirin is one the most used drugs in the world and on the World Health Organization’s list of essential medicines.^8,9^ The main pharmaceutical ingredient of aspirin tablets is acetylsalicylic acid (ASA), a nonsteroidal anti-inflammatory drug, which reduces pain, fever, and/or inflammation in short-term treaments.^8−12^ It is also administered long-term to prevent heart-attacks, ischemic strokes, mini-strokes, and blood clots.^8,10−12^ Thus, extensive efforts have been dedicated to establishing the clinical pharmacokinetics of aspirin: absorption, distribution, metabolism, and excretion. Among those, there is the catalyzed hydrolysis reaction of ASA to salicylic acid (SA), the active form of the drug, which occurs upon absorption in the human body (e.g., in blood, intestinal, or gastric fluids). The ASA solution in water and ethanol (50/50 by volume) has an absorption maximum in the UV region at ∼276 nm.^13,14^ ASA can be made spectrophotometrically active in the VIS region at ∼523 nm through complexation with metal ions such as Fe(III) (Figure 1). Herein, UV–vis absorption spectrophotometry was selected for the quality control based on three key reasons.^5−7^ First, it is widely used in a multitude of applied and fundamental science applications such as monitoring the progress of a reaction and verifying the purity and composition of pharmaceutical drugs (i.e., quality control and quality assurance – QC/QA).^5−7^ Second, it was one of the laboratory skills requested by students for the fine-tuning of instrumentation skills following the COVID-19 academic shut-down. Third, it is one of the instruments recommended by the ACS guidelines for certification in optical molecular spectroscopy.^3,4^

Various undergraduate laboratory courses such as *General Chemistry*, *Organic Chemistry*, and *Analytical Spectroscopy* have intensively explored the synthesis, hydrolysis reaction, and quantification of ASA.^16−24^ Examples of instrumentation-based teaching techniques for the qualitative and quantitative analysis of ASA include chromatography,^16,21^ absorption with portable devices,^17,22,23^ flatbed scanning,^18^ fluorescence emission,^19,23^ Raman,^20,23^ Fourier transform infrared,^24^ and gas chromatography–mass spectroscopies.^24^ However, the kinetics of the hydrolysis reaction of ASA at various temperatures (e.g., reaction order, k, and *E*_a_) has not been examined within a learning, laboratory setting. Furthermore, the molar absorptivity (ε) value of the ASA-derived iron(III) complex was initially estimated using the Beer–Lambert law as no literature value could be determined. In this study, novel elements were incorporated to aid the learning process: (i) the implementation of a well-established pharmaceutical assay (i.e., QC/QA of aspirin) with novel elements of practice for the development of in-depth instrumentation and analytical chemistry skills, (ii) the first-time evaluation of fundamental kinetics aspects of the hydrolysis reaction of ASA in a learning setting, and (iii) a process-oriented guided-inquiry learning (POGIL) to improve research process skills. Utilization of student-centered instruction practices such as POGIL has repeatedly been shown to enhance academic achievement and retention in science and engineering.^25−27^ For example, a Physical Chemistry laboratory experiment was recently developed using a similar POGIL framework for gas-phase infrared (IR) spectroscopy.^27^ In this process, students were provided with a set of guided questions (“data-think cycles”), which encouraged them to independently engage in well-established scientific practices for the successful collection and analysis of the IR spectra (e.g., developing an energy diagram, refining the equations used to model the spectrum, and selecting the spectra resolution). It was concluded that this active learning approach (POGIL) should be emphasized more often in physical chemistry laboratory experiences because it fully engaged the students, who also gained appreciation and understanding of challenging concepts “without getting overwhelmed or bogged down”.^27^ Herein, POGIL was implemented as a Do-It-Yourself approach in the third laboratory component (student-centered), after the students acquired and refined the necessary knowledge and skills to work independently through the first and second laboratory components (instructor-led). Thereby, POGIL is expected to engage students through critical thinking and shift their “thinking like a scientist^”^ concepts from novice to experienced scientists.^25−27^ These constructs are necessary for success in the workforce and civic life, where similar Do-It-Yourself approaches are part of the daily routine and life, when solutions to problems must be independently found and implemented.

## Experimental Overview

### Chemicals

The following materials were purchased from certified vendors: acetylsalicylic acid (ASA, 99% Acros Organics), iron(III) nitrate nonahydrate (Fe(NO_3_)_3_·9H_2_O, Alfa Aesar, 98%), methanol (CH_3_OH, HPLC grade, Fisher Chemical), hydrochloric acid (HCl, certified ACS Plus, Fisher Chemical), sodium hydroxide (NaOH Pellets, Supelco), sodium phosphate monobasic dihydrate (NaH_2_PO_4_·2H_2_O, Fisher Chemical), sodium phosphate dibasic dodecahydrate (Na_2_HPO_4_·12H_2_O, 99% Thermo scientific), and salicylic acid (SA, Lab grade). Aspirin tablets containing 325 mg of ASA each were obtained over the counter: Equate, Certified, and DG Health (Table S1). Deionized (DI) water was utilized throughout all experiments unless otherwise specified. CAS numbers were provided in the Supporting Information.

### Laboratory Component #1: Preparation of Samples and Standards

#### Preparation of 10 mM of Pure ASA stock Solution and Over-the-Counter Aspirin Tablet Solutions

Aqueous solutions of 1 M of HCl and 1 M of NaOH were prepared in water. Each over-the-counter aspirin tablet was weighed and split in half (Table S2). The two halves were weighed and dissolved in water, into separate 125 mL Erlenmeyer flasks. Each solution was then diluted with water, in a 100 mL volumetric flask. Fe(NO_3_)_3_·9H_2_O was weighed out in excess (0.048 g) and transferred to a 10 mL graduated cylinder. Approximately 0.3 mL of the tablet solution was added to the 10 mL graduated cylinder, mixed with Fe(NO_3_)_3_·9H_2_O, and diluted up to 10 mL with water. The pH was adjusted to ∼1.6 using 1 M of HCl.

#### Preparation of ASA Standards and Blank

The following calibration standards (n = 9–10) and a blank were prepared: 0.00, 0.16, 0.20, 0.24, 0.28, 0.32, 0.36, 0.40, 0.44, and 0.48 mM through both serial and parallel dilutions (Tables S3 and S4). The pH was lowered to ∼1.6 with 1 M of HCl for all solutions.

### Laboratory Component #2: Concentration Determinations by UV–Vis Absorption Spectrophotometry

#### Acquisition Parameters

Prelaboratory, students were provided with a detailed standard operating procedure (SOP) for the operation of the Cary 60 absorption spectrophotometer (Supporting Information). Measurements were carried out under the instructor’s supervision using the *Scan* and *Concentration* applications of the Cary WINUV software, which were described within the SOP. Approximately 3 mL of blanks, standards, and sample solutions were placed into optically transparent cuvettes of 1 cm path length and were measured with a spectral resolution of 1.0 nm, in triplicates. Absorption spectra of blank solutions were measured first and were automatically subtracted from those of standards and analyte samples.

#### Concentration Determinations from Beer–Lambert Law

The *Scan* application facilitated the collection of absorption spectra (400–800 nm). After completing the spectrum scan (Figure 1), the absorbance maxima were identified on the spectrum and the corresponding X (peak position in nm) and Y (peak absorbance) values were noted for the concentration calculations using the Beer–Lambert law.

#### Concentration Determinations from Calibration Curves

**Figure 2:**
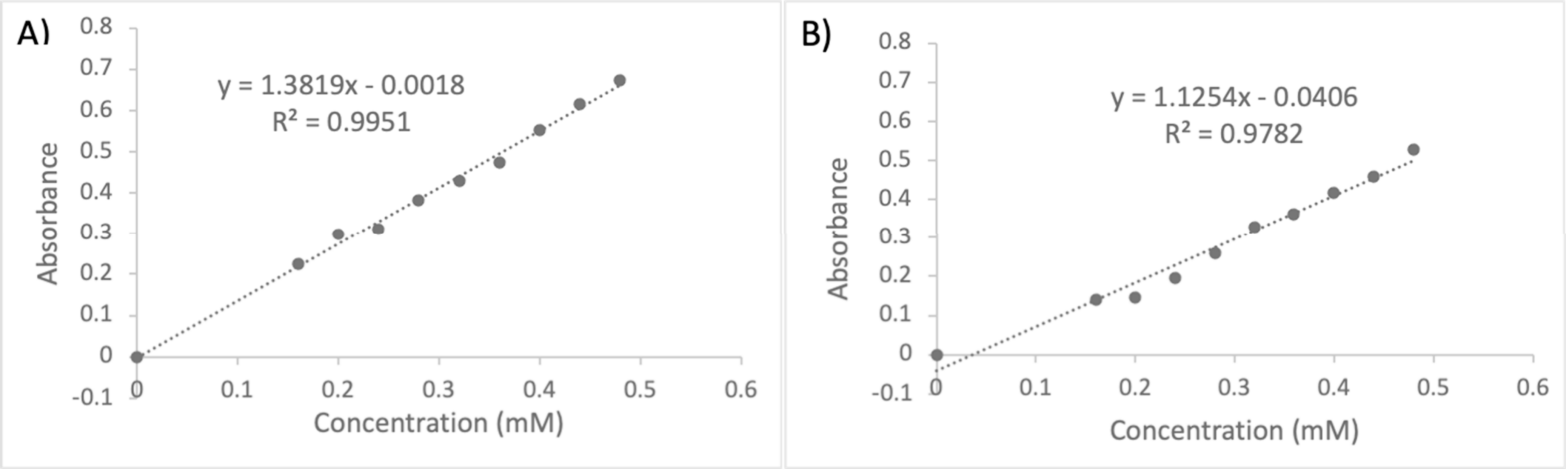
Illustrative external calibration curves constructed by students with *n* = 9 standards and one blank in the 0.00–0.48 mM of ASA concentration range using (A) parallel and (B) serial dilutions.

The *Concentration* application enabled the construction of an external calibration using the prepared standards (Figures 2 and S1). Once constructed, the calibration curve was validated through the *R*^2^ factor, and the unknown concentration of the aspirin analyte was determined through interpolation.

### Laboratory Component #3: Kinetics of Aspirin Hydrolysis

#### Construction of an External Calibration Curve for SA

Students were given the opportunity to further practice the laboratory skills of the first two lab components through the preparation and measurements of n = 9–10 standards from a 0.01 M of SA stock solution using either serial or parallel dilutions (Tables S5 and S6).

#### Hydrolysis Reaction of Aspirin Solution at Different Temperatures (25, 37, 50, 75, and 85 °C) and Constant pH (7.4 ± 0.4)

An ASA solution of 11.1 mM was prepared in 2 mL of methanol, at room temperature, in a 100 mL volumetric flask. Phosphate-buffered saline (PBS) 10x preheated at the desired temperature was then prepared and added up to the 100 mL mark to adjust the pH to a constant 7.4. The resulting mixture was placed in a water bath (25, 37, 50, 75, and 85 °C) and 1 mL aliquots were taken out as a function of time and mixed with 14 mL of PBS before measuring the SA concentration with the *Scan* application (250–350 nm).

#### Concentration Determination from Calibration Curves

The *Concentration* application enabled the construction of an external calibration using the prepared SA standards and the absorption maximum (λ_max_ = 293 nm) identified in the previous *Scan* application. The resulting kinetics data (Table S7) was then evaluated in Excel to establish the overall reaction order, the associated rate constant (k), and the activation energy (*E*_a_).

## Hazards

Both sodium hydroxide (NaOH) and hydrochloric acid (HCl) are highly corrosive to living tissue and many materials. Exposure to these chemicals might cause skin and eye irritation. HCl may also cause respiratory irritation. Dissolution of NaOH is highly exothermic, and the resulting heat may cause burns or ignite flammables. Methanol is a highly flammable liquid and vapor; it is toxic if swallowed, inhaled, or in contact with skin. It may also cause damage to eyes by ingestion. Iron(III) nitrate nonahydrate (Fe(NO_3_)_3_·9H_2_O) is a strong, hygroscopic oxidizer, which can cause eye, skin, and respiratory tract irritations. Thus, all related reactions and preparations must be carefully handled in a chemical fume hood and related personnel protective equipment (PPE) like laboratory coat, goggles, and rubber protective gloves must be used throughout experiments. The standard first aid measure for eye and skin spills includes continuous washing for at least 15–20 min with water.

## Results and Discussion

### Laboratory Component #1: Preparation of Samples and Standards

Before proceeding with the preparation of samples and standards, instructor demonstrations and hands-on practice exercises were implemented to fine-tune fundamental laboratory skills: LS1–weighing, LS2–solution handling, LS3–micropipetting, and LS4–operation of a pH meter. These skills were anonymously evaluated by the course instructors after students’ practice, using a Likert scale from 1 to 5 (Table 1). The Likert scale simply determined if students had competency in a particular technique with 1 corresponding to no level of competence or awareness (the student had no experience in the skill area) and 5 to a high level of competence or expert proficiency (the student had extensive experience in the skill area).^28,29^ The evaluations were completed through observations of each student’s performance during experiments. Variations in LS values (Tables 1, S8–S13) were noticed within each group of chemistry students and between the two groups of students corresponding to different academic years (n = 14 in group #1 and n = 12 in group #2). Descriptive notes were taken by all instructors with respect to each of the five laboratory skills to facilitate the subsequent calibration process of the Likert scores.

**Table 1:** Mean Instructor Ratings and Standard Deviations (SD) of the Laboratory Skills (LS) Observed after the Related Training (n = 14 Students in Group #1 and n = 12 Students in Group #2)^a^

	**Group #1**	**Group #2**
**Laboratory Skill (LS)**	**Mean ± SD**	**Mean ± SD**
LS1		
Balance operation	4.57 ± 1.16	4.29 ± 0.81
LS2		
Storing	4.60 ± 1.20	4.29 ± 0.81
Following Instructions	4.60 ± 1.50	4.46 ± 0.88
Volume Measurement	4.20 ± 1.30	4.21 ± 1.02
LS3		
Contamination	4.40 ± 0.90	4.21 ± 0.78
Pipette Angle	4.90 ± 0.40	3.79 ± 1.35
Storing Pipettes	4.10 ± 1.50	4.58 ± 0.68
Prewetting	4.00 ± 1.52	2.56 ± 1.18
Change Tips	4.60 ± 1.10	5.00 ± 0.00
LS4		
Contamination	4.50 ± 1.46	4.29 ± 0.95
Storage	3.93 ± 1.49	4.83 ± 0.38
LS5		
Setup	3.30 ± 1.60	4.30 ± 0.82
Contamination	3.70 ± 2.00	4.90 ± 0.32
Handling	3.20 ± 2.10	4.70 ± 0.67

a LS1–weighing, LS2–solution handling, LS3–micropipetting, LS4–operation of a pH meter, and LS5–operation of a modern, benchtop UV–vis absorption spectrophotometer. Rating was assigned by instructors on a Likert scale from 1 to 5 based on the students’ level of competency in the observed lab skill. Instructors agreed that a rating of 5 corresponded to a high level of competence or expert proficiency (the student had extensive experience in the skill area), 4 to a moderately high level of competence or advanced proficiency (the student had good experience in the skill area), 3 to an average level of competence or intermediate proficiency (the student had some experience in the skill area), 2 to a low level of competence or basic proficiency (the student had little experience in the skill area), and 1 to no level of competence or awareness (the student had no experience in the skill area).

LS1 consisting of weighing mg quantities of reagents resulted in mean class values of 4.57 and 4.29 with standard deviations (SD) of 1.16 and 0.81 for groups #1 and 2, respectively. The SD values suggest that additional practice with handling an analytical balance (e.g., balance pan spills and improper shutting of glass doors) and weighing boats (e.g., inadequate boat movement during measurements) is needed. LS2 had some of the highest subskill rankings for group #1 with mean values of 4.60 ± 1.20 (storing and transferring liquids) and 4.60 ± 1.50 (following instructions for handling solutions including the preparation of standards). This is likely related to the remedial hands-on experience that students in this group gathered prior to and during this lab. It should be noted that group #1 of students (Fall 2022) was impacted to a larger degree by the COVID-19 pandemic academic shutdown than group #2 (Fall 2024). LS3 associated with micropipetting had high mean values (4.00–4.90) for group #1. Lower LS3 ratings were noticed for group #2 than group #1 regarding the pipetting angle and prewetting subskills (Table 1). Thus, repetitions of micropipetting (1–10 μL) and tip exchange were recommended for further improvement. LS4 associated with measuring pH with a benchtop pH-meter had higher mean values for group #2 than group #1 (e.g., 3.93 ± 1.49 versus 4.83 ± 0.38 for the storage of the pH probe subskill). This is probably due toa single student outlier due to no prior related experience. Additional observations related to the assessment of the LS1-LS5 skills and a brief comparison among the two cohorts of students were provided in Tables S8–S13 and Supporting Information. Because mean values (arithmetic averages – Tables S8–S12) are more sensitive to outliers, which can significantly skew the average, median values (positional averages – Table S13) were also estimated. The median values and corresponding modes are expected to provide a more accurate representation of the central tendency in skewed distributions such as the subskill ratings of LS4, where the student outlier was noted. Indeed, the median and mode values (Table S13) for the LS4 subskills were higher (5.0 and 5 and 5.0 and 5) than the corresponding mean values (4.29 ± 0.95 and 4.83 ± 0.38). Median values and corresponding modes also helped confirm the need of further practice, which was recommended by instructors for the prewetting subskill associated with LS3 (median and mode of 2.0 and 2 versus median ± SD of 2.56 ± 1.18). Percentile scores below the mean (Table S13) identified low to moderate levels of competency and facilitate the subsequent implementation of remedial exercises in group #2, similarly to group #1 (e.g., median of 2.0, mode of 2.0, and percentile of 64.71% below mean for prewetting in LS3). Overall, the in-class demonstrations and hands-on practice gave students in both groups the opportunity to develop and refine skills, which they identified as problematic prior to (group #1) or during (group #2) the implementation of the procedural experiments. This was demonstrated by the mean and median rating values of LS1-LS4, mostly corresponding to very high and high levels of competencies.^28,29^ The LS5 subskills were evaluated within the frame of the laboratory components #2 and 3, where they mostly utilized for the first time (group #1) or refined (group #2). The quality of the student work in laboratory component #1 was then examined through the ASA concentration determinations in the subsequent laboratory component #2.

### Laboratory Component #2: Concentration Determinations by UV–Vis Absorption Spectrophotometry

In this laboratory component, students determined the concentration of ASA in three over-the-counter pharmaceutical formulations of aspirin (*i.e*., Equate, Certified, and DG Health) using a benchtop UV–vis absorption spectrophotometer. This was achieved with the help of two well-known analytical approaches: the Beer–Lambert law (eq 1) and external calibration curves (Figure 2). In the first approach, the Beer–Lambert law relates to the attenuation (I) of the intensity of the incident light (I_0_), at a specific wavelength, to the properties of the analyte material, through which the light is traveling. Briefly, students placed the aqueous aliquots of iron(III) salicylate complex in a cuvette of a known optical path length (l = 1 cm) and recorded its absorbance value (e.g., A = 0.519, Figure 1) at the wavelength maximum (e.g., λ_max_ = 523.5 nm, Figure 1) through a full spectrum *Scan*. A molar absorptivity (ε) value of 1.378 mM^–1^ cm^–1^ was provided for the ASA concentration determinations using the Beer–Lambert law (Supporting Information). The amount of ASA estimated by students for all aspirin tablets had percent differences of 15.47–17.27% with respect to the value listed on the three pharmaceutical brands (325 mg). Larger variations were associated with inaccuracies in transferring of the solid tablets during weighing (LS1), the solution handling (LS2), and the student reading of A values directly from spectral plots (LS5):
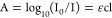
1In the second approach, two external calibration curves (Figure 2) were successfully constructed using nine standards and one blank in the 0.00–0.48 mM of ASA range. This task was completed using the *Concentration* application of the UV–vis absorption spectrophotometer, at the identified absorption maximum of the iron(III) salicylate complex (λ = 523.5 nm from the *Scan* application). In the parallel dilutions, students prepared the standards from a single stock solution, which required additional, remedial practice of skills LS1–LS3. The parallel dilutions helped students identify outliers on their calibration curves and promptly correct the problematic standards. Conversely, outliers on the calibration curve, which students constructed through serial dilutions, required the preparation of all standards following the identified outlier and thereby, additional practice of skills LS1-LS3. Although students preferred the serial dilution due to its speed of implementation, it was more time-consuming when standard(s) had to be rectified. In both cases, a relevant number (n = 9–10) of calibration standards and high determination coefficients, which are typically set as the linearity threshold in chemistry research (R^2^ ≥ 0.995), were obtained because of the remedial measures. Once the calibration curves were constructed, students determined the ASA concentration of the provided over the counter aspirin tablets through interpolation (Supporting Information). The percent differences with respect to the provided value of active pharmaceutical ingredient (325 mg) were 1.1–35.3%. According to the U.S. Pharmacopeia, an aspirin tablet must contain no less than 90% and no more than 110% of the active pharmaceutical ingredient.^30^ Because a few student results deviated by more than ±10% of ASA, additional laboratory practice (skills LS1-LS5) was recommended for the accurate quantification of ASA in the measured tablets. Table 1 shows lower mean LS5 values for group #1 than group #2. This was attributed to the very limited to no experience in the skill area for group #1 of students pre- and during the pandemic. LS5 notes the students’ ability to operate the UV–vis absorption spectrophotometer correctly. The most challenging aspect of LS5 for the students in group #1 was the setup of cuvettes for multiple standards. However, after the second trial, students were more confident with the instrumentation procedure and there was significant improvement in the LS5 subskills. Group #2 of students performed very well in the operation of a modern, lab benchtop UV–vis absorption spectrophotometer (LS5 mean values of ≥4.30 and SD ≤ 0.82). Several students in this class had extensive hands-on experience that was acquired postpandemic through undergraduate research projects in research active laboratories.

### Laboratory Component #3: Kinetics of Aspirin Hydrolysis

**Figure 3:**
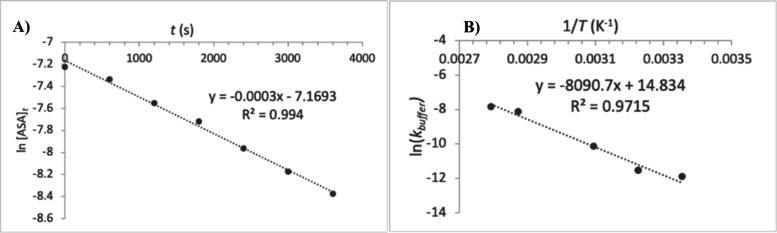
(A) Illustrative kinetics plot of the ln [ASA]_*t*_ as a function of time (t in s), at 75 °C. Data were collected every 10 min, over a 60 min time interval (total of *n* = 7 data points). (B) Illustrative Arrhenius graph of ln(*k*_buffer_) versus the reciprocal of temperature (1/T in K^–1^) at 25, 37, 50, 75, and 85 °C (total of *n* = 5 data points).

This laboratory component built upon students’ theoretical knowledge of the kinetics of reactions and the hands-on skills (LS1–LS5) acquired in the prior two laboratory components. Briefly, students examined the k and *E*_a_ values of the hydrolysis reaction of ASA under pseudo-first order conditions, at different temperatures. The reaction is typically catalyzed under acidic (pH < 2.4) or basic (pH > 8.5) conditions (eq 2).^15^ Herein, students transformed the second order reaction into a pseudo-first order reaction (eq 3) by completing it in a buffer solution, at 7.4 ± 0.4 (i.e., at constant hydroxide concentration). This reduced the complexity of the kinetics experiments and simplified the calculations of the reaction rate constants (k) and the corresponding activation energy (*E*_a_).
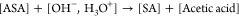
2
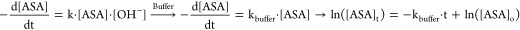
3A physiological pH was selected to encourage students to reflect on the pharmacokinetics aspects of the ASA hydrolysis within the human body. The concentration of ASA was determined by difference from the concentration of its SA product (Supporting Information). The SA concentrations were estimated using the SA peak at ∼293 nm and an external calibration curve (n = 9–10 standards and a blank), which students constructed through serial or parallel dilutions, similarly to the process learned in lab components #1 and 2 for ASA (Supporting Information). The buffered hydrolysis of ASA was carried out at different temperatures (25, 37, 50, 75, and 85 °C), within a 1 h time interval, and aliquots were measured every 10 min to determine the time-dependence of the SA concentrations at each temperature. Students then calculated the ln[ASA]_t_ values from the measured [SA] concentrations and plotted them as a function of time (Figure 3A). The slope of the linear equation (R^2^ = 0.994) associated with this kinetics plot at each temperature was identified as the −*k*_buffer_ value (eq 3) of the pseudo-first order reaction. For the illustrative plot at 75 °C (Figure 3A), *k*_buffer_ was estimated to be ∼3.0 x10^–4^ s^–1^. The calculated *k*_buffer_ values at each temperature were: 7.0 × 10^–6^ s^–1^ at 25 °C, 1.0 × 10^–5^ s^–1^ at 37 °C, 4.0 × 10^–5^ s^–1^ at 50 °C, 3.0 × 10^–4^ s^–1^ at 75 °C, and 4.0 × 10^–4^ s^–1^ at 85 °C. A similar rate constant of 4.5 × 10^–6^ s^–1^ was reported in literature for the aspirin degradation in solution at 25 °C.^31,32^ The students noted that the higher the temperature, the larger the rate constant value, *k*_buffer_, and the faster the reaction. This facilitated the discussion of the Arrhenius eq (eq 4) and the temperature dependence of the rate constant. Finally, students constructed the Arrhenius plot to find *E*_a_ (Figure 3B). Using the resulting linear equation of the ln *k*_buffer_ versus 1/T, students estimated the absolute value of *E*_a_/R to be ∼8091 K. (Figure 3B). From here, *E*_a_ was calculated to be ∼67.3 kJ mol^–1^ for the buffered hydrolysis of ASA in a PBS solution at 7.4 ± 0.4 (eq 4, R of 8.314 J/(mol K)). No reference *E*_a_ data could be determined in literature at this pH.
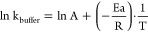
4

### Pre- and Post-laboratory Surveys

Survey questions (SQ #1–3 in Table 2) were administered anonymously to assess the overall laboratory experience and interest pre- and postcompletion (Institutional Review Board IRB-2014–1114). The student ratings were on a scale from 1 to 10, with 10 being the highest value (i.e., the most positive feedback). Prelaboratory, students’ interest in the instrumentation-based laboratory work was high (8.2), but it increased postlaboratory (8.8) as also suggested by the received comments: “*I took Gen Chem II during covid, and we did not have any [hands-on] laboratories. I learned a lot from you, and I really appreciated your effort*.” The overall experience was very positive, with an average of 9.5 and a low standard deviation of 0.7. The survey showed the high enthusiasm and appreciation students had for this hands-on laboratory on modern instrumentation: “*I enjoyed the labs and learned a lot about instrumentation*.”

**Table 2:** Mean Class Values (n = 14–the First Cohort of Students) And Standard Deviations (SD) for the Overall Experience and Interest Students Showed in This Laboratory Module^a^

**Survey Question (SQ)**	**Mean ± SD**
SQ#1: Overall interest in the proposed experiments prelaboratory	8.2 ± 1.7
SQ#2: Overall interest in the proposed experiments postlaboratory	8.8 ± 1.3
SQ#3: Overall experience in this laboratory	9.5 ± 0.7

a The survey questions (SQ) were administered anonymously pre- and post-laboratory.

In the last laboratory component, students explored key pharmacokinetics aspects of the ASA hydrolysis reaction within a POGIL frame. None of the students had prior experience with POGIL so discussions were carried out based on illustrative examples and related reading material. Afterward, students designed and conducted the POGIL-based laboratory activities, while the instructor acted only as a facilitator of self-discovery, assessed progress and addressed preparatory questions.^23−25^ Herein, POGIL was completed by the students in the three steps: (1) exploration - students formulated the kinetics questions (“What are the k and *E*_a_ values for the ASA hydrolysis reaction?”) and a hypothesis (“The buffered ASA hydrolysis is a pseudo-first order reaction”), (2) concept invention - students constructed an experimental plan to conduct the scientific discovery (see methodology description for the third laboratory component in Supporting Information), and (3) application - students implemented the proposed plan to address the kinetics questions and the hypothesis. Students worked as a team, and individual students in the team were assigned specific roles such as time and task manager, data collector, and presenter. According to the feedback received, this student-centered instruction was the most valuable learning experience: *“I find these inquiry labs difficult at first but much more meaningful”* and *“I think we should give more Inquiry-based learning labs in college it allows the student to work through the experiment at hand, requiring critical thinking and problem solving*.*”* This learning method helped refine students’ research process skills including the ability of applying previously accumulated knowledge, information processing, problem solving, creative thinking, teamwork, metacognition, and self-assessment.

### Post Laboratory Assessment

The laboratory experience culminated with the preparation of a final laboratory report by each student. Each of the three-laboratory components were part of this summative assessment, and a related grading rubric in value of 100 points total is presented in the Supporting Information together with the specific report questions, representative data collection tables (Tables S14–S16), a tentative timeline (Table S17), and answer keys for each laboratory component. Illustrative examples were selected from the class responses. The average class score corresponded to a B letter grade (86–87%), and further confirmed the successful achievement of the proposed student learning goals.

## Conclusions

An undergraduate laboratory experiment dedicated to the kinetics of the aspirin hydrolysis reaction and its concentration determination from calibration curves of a relevant number of standards (n = 9–10) was successfully implemented. This helped finetune both basic and in-depth laboratory skills and encouraged critical thinking. All students completed the assigned tasks, and instructors have unanimously agreed that students in both groups were competent (≥88%) in laboratory skills LS1-LS5, but additional hands-on practice was recommended for LS5 (group #1) and LS3 (group #2). The three components (3 h each) of this new *Physical Chemistry* laboratory module are flexible and easily adaptable to other science curricula in *Instrumental Analysis, Pharmaceutical Chemistry, Biochemistry*, and *Forensic Chemistry*. The formative assessments demonstrated that the hands-on learning activities were well received by the junior and senior chemistry students. Research process skills were particularly stimulated by the POGIL in the third, kinetics laboratory component, which students described as engaging and beneficial to their professional development.

## Supporting Information Available

Additional material for students and instructors, including material for setting up, adapting, and carrying out the laboratory module in other academic settings, student handouts, instructor notes, detailed experimental procedures, safety information and CAS numbers, pre- and postlab assessments, and representative data with sample calculations for instructors (PDF, DOCX)
