# Supplementary material for: A Laboratory Module for Physical Chemistry and Analytical Chemistry: The Kinetics of Aspirin Hydrolysis and Its Quantitation in Pharmaceutical Tablets
Source: J Chem Educ. 2025 Jan 31;102(2):746–53. doi: 10.1021/acs.jchemed.4c00809 (PMC11823444; doi:10.1021/acs.jchemed.4c00809)
Supplement: Supplementary file 1 — ed4c00809_si_001.pdf [file ed4c00809_si_001.pdf]

<u>**SUPPORTING INFORMATION**</u>

A laboratory module for Physical Chemistry and Analytical Chemistry: The kinetics of aspirin hydrolysis and its quantitation in pharmaceutical tablets

Victoire Delattre,[1] Remi Olivier Labeille,[1] Nicholas Slade Shropshire,[1] Kyra Grace Kaiser,[1] Brent Kirkland,[1] Keith Zvoch,[2] and Ioana Emilia Pavel[*]

[1]Department of Physical and Environmental Sciences, College of Science, Texas A&M University at Corpus Christi, 6300 Ocean Drive, Corpus Christi, Texas, 78412-5800

[2]Department of Education Studies, College of Education, University of Oregon, 1215 University of Oregon, Eugene, OR 97403-1215

*Corresponding author: ioana.pavel@tamucc.edu

I. STUDENT NOTES

1. Supplies provided to each lab station (2-3 students)

Each lab station was provided with the following supplies for conducting the experiments: 2 mL of methanol (CH_3OH, HPLC grade, CAS: 67-56-1, Fisher Chemical), 5 mL of 1 M of hydrochloric acid (HCl, certified ACS Plus, Fisher Chemical, CAS: 7647-01-0), 30 mL of 1 M of sodium hydroxide (NaOH Pellets, Supelco, CAS: 1310-73-2), 0.5 g of iron (III) nitrate nonahydrate ($Fe(NO_3)_3$•$9H_2O$, Alfa Aesar, 98%, CAS: 7782-61-8), 3.169 g of sodium phosphate monobasic dihydrate (NaH_2PO_4•$2H_2O$, Fisher Chemical, CAS: 1372-35-0), 28.55 g of sodium phosphate dibasic dodecahydrate (Na_2HPO_4•$12H_2O$, 99% Thermo scientific, CAS: 10039-32-4), potassium hydroxide (KOH, Fisher Chemical, CAS: 1310-58-3), 200 mg of acetylsalicylic acid (ASA, 99% Acros Organics, CAS: 50-78-2), 0.13812 g of salicylic acid (SA, Lab grade, CAS: 69-72-7), one spatula, one glass rod, 5-10 weigh boats, 100-1,000 µL micropipettes, one 200-mL volumetric flask, one 250-mL volumetric flask, two 125-mL Erlenmeyer flasks, four 100-mL volumetric flasks, glass stopers for volumetric flasks, one marker, one 10-mL graduated cylinder, two funnels for 100-mL volumetric flasks, five 20-mL glass vials, parafilm, 3-mL cuvettes (340-750 nm and 285-750 nm), labeling tape, a calibrated pH meter, one stirring hot plate, a thermometer, magnetic stir bars, stopper, a water bath or 500-mL beaker filled with water, labeled waste containers, over-the-counter aspirin tablets that were used before the expiration date, high purity water (18 MΩ cm), deionized (DI) water, and a water bottle. Table S1 summarizes the information collected from the package labels of the over-the-counter aspirin tablets.

Tables S1. Information about the over-the-counter aspirin tablets. Acetylsalicylic acid (ASA) in mg refers to the amount of active pharmaceutical ingredient.

**Brand**	**Distributor**	**Vendor lot**	**ASA amount (mg)**
Certified	LNK International Inc.<br>60 Arkay Drive, Hauppauge, NY 11788	eBay<br>P123959	325
DG Health	Dolgencorp., LLC<br>100 Mission Ridge, Goodlettsville, TN 37072	Dollar General<br>P125414	325
Equate	Walmart Inc.<br>Bentonville, AR 72716	eBay<br>1FE2281A	325

2. Safety and hazards

All glassware must be rigorously cleaned and checked for any potential damage before use. Sodium hydroxide (NaOH) and hydrochloric acid (HCl) are highly corrosive to living tissue and many materials. Exposure to these chemicals might cause skin and eye irritation. HCl may also cause respiratory irritation. Dissolution of NaOH is highly exothermic, and the resulting heat may cause burns or ignite flammables. Methanol (CH_3OH) is a highly flammable liquid and vapor. It is toxic if swallowed, inhaled, or in contact with skin. It may also cause eye damage. Iron (III) nitrate nonahydrate ($Fe(NO_3)_3 \cdot 9H_2O$) is a strong, hygroscopic oxidizer, which can cause eye, skin, and respiratory tract irritations. Aspirin (ASA) may be harmful if taken without a prescription; it may cause skin, eye, and respiratory irritation. Salicylic acid (SA) may cause severe eye, skin, and respiratory tract, and gastrointestinal irritations with nausea, vomiting, and diarrhea. SA may also have negative effects on the reproductive system and fetuses. All reactions and sample preparations must be carefully handled in a clean chemical fume hood and personnel protective equipment (PPE) such as laboratory coat, goggles, and rubber protective gloves must be used throughout all experiments. The standard first aid measure for eye and skin spills includes continuous washing for at least 15-20 min with water.

3. Experimental procedures for students

<u>Laboratory Component #1: Preparation of samples and standards</u>

Preparation of a 10 mM of ASA stock solution

Pure ASA powder will be dissolved in 1 M of NaOH and diluted with DI water to 100 mL, in a chemical fume hood, following the procedure given below. The stock solution is considered fresh for approximately 38 hr.

1. Weigh out 0.1802 g of ASA and transfer it to a 125 mL Erlenmeyer flask.
2. Add 5 mL of 1 M of NaOH to the ASA Erlenmeyer flask and gently swirl.
3. Once ASA is dissolved, transfer it to a 100 mL volumetric flask.
4. Dilute the solution up to the 100 mL mark with DI water. Insert a stopper and mix the solution by inversion and shaking or using a stir bar on a stirring plate. The final pH is ~12.2.

Preparation of over-the-counter aspirin tablet solutions

1. Measure the mass of the whole aspirin tablet and record it in Table S2. Then, prepare the aspirin tablets for measurement by gently splitting an aspirin tablet in equal halves with a spatula, directly into the weight boat. Weigh the two halves separately and record the mass in Table S2. Mark the two weight boats with #1 and #2.

Note: Aspirin tablets of regular dose (325 mg of ASA) are split into halves to facilitate the use of a single external calibration curve for various brands (regular versus low dose). Low dose aspirin formulations (81 mg of ASA) should not be split in half and 0.6 mL of the tablet solution should be added instead of 0.3 mL.

2. Add each half of the aspirin tablet to a separate 125 mL Erlenmeyer flask and mark the flasks with #1 and #2. Pipette out 5 mL of 1 M of NaOH from a graduated cylinder directly into the flask and gently swirl the mixture. Let sit for 15 minutes with intermittent swirling.
3. After dissolving each half tablet, transfer this aspirin solution to a 100 mL volumetric flask using a funnel. Rinse the walls of the Erlenmeyer flasks with DI water and transfer this wash content to the 100 mL volumetric flask before the final dilution with DI water, up to 100 mL mark. Cover with parafilm and mix the solution by inversion and shaking or using a magnetic stir bar. Measure the pH of the resulting solution and record it in Table S2.

4. Weigh out 0.048 g of $Fe(NO_3)_3 \cdot 9H_2O$ on a weighing boat, for each aspirin half tablet and record the measured mass in Table S2. Rinse the mass of $Fe(NO_3)_3 \cdot 9H_2O$ with approximately 5 mL of DI water or less into a 10 mL graduated cylinder and mix it with a long glass rod. Mark each weigh boat and flask with #1 or #2. Add 0.3 mL of each half of the ASA tablet solutions using a micropipette to the graduated cylinder containing the yellow $Fe(NO_3)_3 \cdot 9H_2O$ solution. Once it is dissolved, dilute with DI water up to the 10 mL mark of the graduated cylinder and mix well using a glass rod. The mixture should be violet in color.
5. Pour the contents of the graduate cylinders into 20 mL individual glass vials marked with #1 and #2. Adjust the pH of the resulting solutions to approximately 1.6 using 400 µL of 1 M of HCl and a micropipette. Swirl for uniform mixing before measuring the pH with a pH meter. Record the pH and the used volume of HCl in Table S2.

Table S2. Data collection for the preparation of over-the-counter aspirin tablet solutions.

**Aspirin ___________ (tablet brand) and amount of ASA__________ in mg**	**Whole aspirin tablet**	**1st Half tablet**	**2nd Half tablet**
Mass (g) of aspirin tablets			
pH of aspirin solution before $Fe(NO_3)_3 \cdot 9H_2O$ addition	X		
Mass of $Fe(NO_3)_3 \cdot 9H_2O$ (g)	X		
pH of aspirin solution after $Fe(NO_3)_3 \cdot 9H_2O$ addition	X		
Volume of HCl added (µL)	X		
Volume of NaOH added (µL)	X		

Preparation of ASA standards and blank using parallel dilutions

A blank solution (0 mM of ASA) and six to nine standards (0.480, 0.440, 0.400, 0.360, 0.320, 0.280, 0.240, 0.200, and 0.160 mM of ASA) will be prepared from a single stock solution of ASA (10 mM) through direct, parallel dilutions, using appropriate micropipettes (100-1,000 µL range).

1. Add 0.480 mL of the ASA stock solution to a 10 mL graduated cylinder to prepare the first standard (0.480 mM of ASA), measure and record its pH in Table S3.

2. Weigh out 0.048 g of $Fe(NO_3)_3•9H_2O$ on a weighing boat and record the measured mass in Table S3. Rinse it into the graduated cylinder containing the stock solution with DI water and stir the mixture using a long glass rod.
3. Dilute with DI water up to the 10 mL mark of the graduated cylinder.
4. Pour the contents of the graduate cylinders into 20 mL individual glass vial.
5. Repeat the above steps #1-4 for the remaining standards by using 0.440 mL, 0.400 mL, 0.360 mL, 0.320 mL, 0.280 mL, 0.240 mL, 0.200 mL, and 0.160 mL of ASA stock solution.
6. Adjust the pH of the resulting solutions to ~1.6 pH with 400 µL of 1 M of HCl and mix well before measuring the pH with a pH meter. Record the pH value in Table S3.
7. Prepare a blank of $Fe(NO_3)_3•9H_2O$ solution following the above steps including the pH adjustment, but without adding ASA stock solution (0 mM).

Table S3. Data collection for the preparation of ASA standards and blank using parallel dilutions.

**Preparation of standards**	**Blank**	**Standard #1**	**Standard #2**
pH of aspirin solution before $Fe(NO_3)_3•9H_2O$ addition			
Mass of $Fe(NO_3)_3•9H_2O$ (g)			
pH of aspirin solution after $Fe(NO_3)_3•9H_2O$ addition			
Volume of HCl added (µL)			
pH after HCl addition			

Note: Add more columns as needed in Table S3 for each ASA standard.

Preparation of ASA standards and blank using serial dilutions

Serial dilutions will be performed from a 0.480 mM solution of ASA down to 0.160 mM, in increments of 0.040 mM. The 0.480 mM solution of ASA (~50 mL) will be prepared from the same stock solution of 10 mM of ASA.

1. Add 2.4 mL of 10 mM of ASA stock solution to a 100 mL beaker.
2. Weigh out 0.048 g of $Fe(NO_3)_3•9H_2O$, add it to the larger beaker from previous step #1 and mix it in using a glass rod. The solution will turn dark purple.
3. Dilute the solution from the above step #2 with 47.6 mL of DI water to prepare a total volume of 50 mL of ASA standard of a concentration of 0.480 mM. This standard will be violet in color.

4. To prepare the other eight (8) standards, calculate in a similar manner the volumes of previous ASA standard and DI water, which are needed for subsequent serial dilutions. Use the dilution rule from Equation 1:

$$M_i \times V_i = M_f \times V_f \quad (1)$$

where i and f refer to the initial and final values, respectively, of the molarity concentration (M in mmol L^{-1}) and volume (V in mL) values.

Note: Fill in Table S4 when calculating the needed volumes for serial dilutions.

5. Once all standards are prepared, transfer them to individual 20 mL glass vials.
6. Adjust the pH of each standard to ~1.6 using 400 µL of 1 M of HCl. Make sure you mix well the standard solutions before measuring the pH with a pH meter.
7. Prepare a blank of $Fe(NO_3)_3$•$9H_2O$ solution (no ASA) following the procedure described in Part III.

Table S4. Data collection for the preparation of ASA standards and blank using serial dilutions.

**Molarity of standard (mM)**	**Volume of standard (mL)**	**Volume of DI water (mL)**	**Volume of HCl (µL)**
0.480	2.40	50.00 – 2.40 = 47.60	
0.440			
0.400			
0.360			
0.320			
0.280			
0.240			
0.200			
0.160			

Waste and cleanup: Clean up the workstations and dispose of all waste as instructed. Put away the lab tools and wash the glassware according to the lab standard procedures and instructor's directions.

Laboratory Component # 2: Concentration determinations by UV-Vis absorption spectrophotometry

ASA concentration determinations from the Beer-Lambert Law

1. Turn on the Cary 60 UV-VIS absorption spectrophotometers following the standard operation protocol (SOP) of the instrument and allow it to calibrate for about 3 min. The SOP file (Cary 60_SOP.pdf) was provided as part of the prelab material.
2. Add 3 mL of the blank solution to a disposable cuvette of 1-cm path length through micro pipetting. Gently wipe off the cuvette walls using Kimwipes and place the cuvette inside the Cary 60 system as instructed in the SOP.
3. Use the ***Scan*** application in the Cary WINUV folder to record a spectrum in 400-800 nm range. This will facilitate the indirect determination of the ASA concentration using the Beer-Lambert Law. Setup the parameters for measuring the ASA blank following the SOP and measure the blank by pressing Zero. Remove the cuvette from the instrument.
4. Add 3 mL of the ASA standard (0.320 M) to a disposable cuvette of 1-cm path length, place it into the cuvette holder of the Cary 60 system, and measure it following the same SOP by pressing Start.
5. Remove the ASA standard solution and save the UV-VIS absorption spectrum file (ASCII format as .csv file) as instructed in the SOP. Record the maximum absorption wavelength for the subsequent calculation and the calibration curve measurements.

Wavelength maximum for the ASA standard: λ_{max} = ________nm

Absorbance value at λ_{max}: A= _________(unitless)

ASA concentration determinations from the external calibration curve

1. Add 3 mL of the ASA blank solution to a disposable cuvette of 1-cm path length through micro pipetting. Gently wipe off the cuvette walls using a Kimwipe and place the cuvette inside the Cary 60 system for measurement following the provided SOP.
2. Use the ***Concentration*** application in the Cary WINUV folder to directly determine the ASA concentration from the external calibration curve through interpolation. Setup the parameters (e.g., Wavelength maximum of _____ nm, Calibrate During Run using mmol/L, Standards 6-10, and Samples 3-6) for measuring the blank, the ASA standards, and the aspirin tablet solutions following the SOP. Do not forget to input the concentration

values of your standards before proceeding with the measurements. Press Zero to launch the measurement of the Blank.

3. Once the measurement of the ASA blank is completed, replace the ASA blank cuvette with the cuvette containing the lowest ASA concentration of standard. Press Start to proceed with the ASA standard measurements. You will be prompted to give a file name for the calibration curve and to repeat the procedure for the subsequent standards.
4. Once a reliable calibration curve is constructed ($R^2 \geq 0.995$) and saved (.csv file), you will be prompted to insert and measure the ASA tablet samples. The corresponding absorbance and concentration values for each ASA sample solutions are automatically saved in the calibration curve file as .csv file. The instructor will share all the files with the students via e-mail.

Note: Do NOT use a personnel memory stick for collecting the data to avoid infecting the Cary 60 instrument with a computer virus.

5. Close the software and turn off the Cary 60 system.

Waste and cleanup: Clean up the workstations and dispose of all waste as instructed. Put away the lab equipment and wash the glassware according to the lab standard procedures and instructor's directions.

<u>Laboratory component #3: Kinetics of aspirin hydrolysis</u>

Preparation of a 10x phosphate-buffered saline solution (PBS, pH of 7.4) at room temperature

1. Pour 1 L of high purity water (18 MΩ cm) into an appropriate container.
2. Measure out 3.169 g of sodium phosphate monobasic dihydrate and 28.55 g of sodium phosphate dibasic dodecahydrate. Add these amounts to the buffer container containing the water and shake vigorously until dissolved.
3. Determine the pH of the solution using a pH meter. If the pH is slightly above 7.4, slowly add 1 M of HCl to adjust the pH. If the pH is slightly below 7.4, use one 1 M of KOH to modify it to 7.4.

Note: Do NOT forget to mix well the solution before measuring the pH.

4. Label the buffer container (student initials, date, 10x PBS, pH of 7.4).

Preparation of a 10 mM of SA stock solution

Pure SA powder will be dissolved in pure methanol and diluted to 100 mL with 10x PBS solution (pH of 7.4) at room temperature following the procedure given below. The SA stock solution is considered fresh for approximately 38 hr.

1. Weigh out 0.138 g of SA and transfer it to a 100 mL volumetric flask.
2. Add 2 mL of pure methanol to the SA Erlenmeyer flask and gently swirl. Dilute the solution up to the 100 mL mark with 10x PBS. Insert a stopper and mix the solution by inversion and shaking or using a stir bar on a stirring plate.

Preparation of blank and SA standards using parallel dilutions

Parallel dilutions will be completed by pipetting and mixing the designated quantities (Table S5) into 20 mL glass vials.

Table S5. Data collection for the preparation of blank and SA standards using parallel dilutions.

**Concentration (mM)**	**Volume (µL) of 10 mM of SA stock**	**Volume (µL) of PBS solution**
0.107	53.0	4,947
0.222		
0.333		
0.444		
0.556		
0.667		
0.778		
0.867		
1.000		

Preparation of blank and SA standards using serial dilutions

An external SA calibration curve will be prepared through serial dilutions using = 9-10 standards in the 0.107 mM to 1.000 mM concentration range and one blank. These dilutions will be completed using a SA stock solution of 10 mM of SA (pH of 7.4 ± 0.25) and PBS. Procedural data will be collected using Table S6.

1. Label ten (10) glass vials of 20 mL each: one as blank and the rest as standards #1-9.
2. Add 10 mL of PBS to the blank.
3. Record the initial mass of each vial after labeling.
4. To vial #1, add 18 mL of PBS solution then add 2 mL of SA stock. Mix well the resulting solution and record the mass.
5. To vial #2, add 15 mL of solution from vial #1 and 5 mL of PBS solution.
6. Repeat this sequence of steps for all other vials.

Table S6. Data collection for the preparation of the blank and SA standards using serial dilutions. SA refers here to the standard solution taken from the preceding vial.

**Vial #**	**Volume of SA added (mL)**	**Final concentration of SA (mM)**
Blank		
1		
2		
3		
4		
5		
6		
7		
8		
9		

SA spectrum scan

1. Turn on the Cary 60 UV-VIS absorption spectrophotometers following the standard operation protocol (SOP) of the instrument and allow it to calibrate for about 3 min. The SOP file (Cary 60_SOP.pdf) was provided as part of the prelab material.
2. Add 3 mL of the blank solution to a disposable cuvette of 1-cm path length through micro pipetting. Gently wipe off the cuvette walls using Kimwipes and place the cuvette inside the Cary 60 system as instructed in the SOP.
3. Use the ***Scan*** application in the Cary WINUV folder to record a spectrum in 200-800 nm range. This will facilitate the indirect determination of the SA concentration using the Beer-

Lambert Law. Setup the parameters for measuring the blank following the SOP and measure the blank by pressing Zero. Remove the cuvette from the instrument.

4. Add 3 mL of the SA standard to a disposable cuvette of 1-cm path length, place it into the cuvette holder of the Cary 60 system, and measure it following the same SOP by pressing Start.
5. Remove the SA standard solution and save the UV-VIS absorption spectrum file (ASCII format as .csv file) as instructed in the SOP. Record the maximum absorption wavelength for the subsequent calculation and the calibration curve measurements.

Wavelength maximum for the SA standard: λ_{max} = ________nm

Absorbance value at λ_{max}: A= _________(unitless)

SA concentration determination from the external calibration curve

1. Add 3 mL of the SA blank solution to a disposable cuvette of 1-cm path length through micro pipetting. Gently wipe off the cuvette walls using a Kimwipe and place the cuvette inside the Cary 60 system following the SOP.
2. Use the ***Concentration*** application in the Cary WINUV folder to directly determine the SA concentration from the external calibration curve through interpolation. Setup the parameters (e.g., Wavelength maximum of _____ nm, Calibrate During Run using mmol/L, Standards 6-10, and Samples 3-6) for measuring the blank and SA standards following the SOP. Do not forget to input the concentration values of your standards before proceeding with the measurements. Press Zero to launch the measurement of the Blank.
3. Once the measurement of the SA blank is completed, replace the SA blank cuvette with the cuvette containing the lowest SA concentration of standard. Press Start to proceed with the standard measurement. You will be prompted to give a file name for the calibration curve and to repeat the procedure for the subsequent standards.
4. Once a reliable calibration curve is constructed ($R^2 \geq 0.995$) and saved (.csv file), you will be prompted to insert and measure the SA samples. The corresponding absorbance and concentration values for each SA solutions will be automatically saved in the calibration curve file as .csv file. The instructor will share all the files with the students via e-mail.

Hydrolysis reaction of aspirin (ASA) at different temperatures (25, 37, 50, 75, and 85°C) and constant pH (7.4±0.4)

1. Add approximately 500 mL of water to a 2,000 mL beaker. Preheat this water bath on a hot plate, at 37°C.
2. Pour 120 mL of PBS solution into a 200 mL volumetric flask. Place the flask in the constant temperature bath and allow the solution to reach 37°C.
3. Weigh out 200 mg of ASA then transfer the powder to an empty 100 mL volumetric flask.
4. Add 2.0 mL of pure methanol to the 100 mL volumetric flask and swirl the flask until the ASA powder has dissolved completely.
5. Add the 37°C buffer solution to the 100 mL volumetric flask. After filling the flask about halfway with the PBS, cover with a stopper and invert the flask several times to ensure proper mixing. Then proceed to fill with PBS until the mark and invert the flask several times.

Note: Using parafilm instead of a stopper might result in melting of the parafilm, especially at higher temperatures.

6. Uptake 1 mL of sample from the volumetric flask and transfer it to a 20 mL sample container with a lid. Add 14 mL of buffer solution of room temperature, mix the contents, and label the container as follows: 0 minutes, 37°C, ASA solution, current date, and student initials.
7. Repeat step 6 and label the subsequent samples with the appropriate time for the kinetics reaction. Proceed with the measurements of these samples every 10 min for a total of 60 min using the Cary 60 UV-VIS absorption spectrophotometer (see steps #9-14 for instructions).

Note: It is advisable to distribute the team tasks: one student should prepare the samples (see instructions of this section**)** and one student should measure the samples (see instructions of the next section).

8. Repeat the procedural steps #1-7 for the following reaction temperatures: 25, 50, 70, and 85°C.

Note: It is not necessary to have a bath temperature of 25 °C, as this is the room temperature.

9. Turn on the Cary 60 UV-VIS absorption spectrophotometers following the standard operation protocol (SOP) of the instrument and allow it to calibrate for about 3 min. The SOP file (Cary 60_SOP.pdf) was provided as part of the prelab material.
10. Add 3 mL of the heated buffer solution to a disposable cuvette of 1-cm path length through micro pipetting. Gently wipe off the cuvette walls using Kimwipes and place the cuvette inside the Cary 60 system as instructed in the SOP.
11. Use the ***Scan*** application in the Cary WINUV folder to record a spectrum in 200-800 nm spectral range. Setup the parameters for measuring the buffer blank following the SOP and measure the blank by pressing Zero. Remove the cuvette from the instrument.
12. Add 3 mL of the kinetic sample to a disposable cuvette of 1-cm path length, place it into the cuvette holder of the Cary 60 system, and measure it following the same SOP by pressing Start.
13. Repeat steps #11 and 12 for each of the remaining samples.
14. Remove the samples and save the UV-VIS absorption spectrum file (ASCII format as .csv file) as instructed in the SOP. Record the maximum absorption wavelength for the subsequent calculation.

Waste and cleanup: Clean up the workstations and dispose of all waste as instructed. Put away the lab equipment, turn off the Cary 60 instrument following the SOP instructions, and wash the glassware according to the instructor's directions.

II. INSTRUCTOR NOTES

1. Pre-laboratory assessments

Illustrative pre-laboratory questions are given below for each laboratory component #1-3. These pre-laboratory assessments can be tailored to the student audience and can be utilized as a remediation tool in preparation for the subsequent laboratory work and the data analysis.

<u>*Laboratory Component #1:*</u>

Calculate the molarity and molality of 4 g of ASA in 10 mL of aqueous solution. The molecular weight of ASA is 180.158 g/mol. Consider 1 g mL^{-1} for the density of water to ease the calculation.

Answer key (Equations 1-4):

$$\text{number of moles (n)} = \frac{\text{mass (m)}}{\text{molecular weight (MW)}} = \frac{4\text{ g}}{180.158\text{ g/mol}} = 0.022\text{ mol} \quad (1)$$

$$\text{Molarity} = \frac{\text{n of solute}}{\text{V of solution}} = \frac{0.022\ \text{mol}}{0.01\ \text{L}} = 2.2\ \frac{\text{mol}}{\text{L}} \qquad (2)$$

$$\text{Mass of solvent (kg)} = \text{density} \times \text{volume} = (1\ \text{g mL}^{-1}) \times (10\ \text{mL}) = 10\ \text{g} = 0.01\ \text{kg} \ (3)$$

$$\text{Molality} = \frac{\text{n of solute}}{\text{mass of solvent in kg}} = \frac{0.022\ \text{mol}}{0.01\ \text{kg}} = 2.2\ \frac{\text{mol}}{\text{kg}} \qquad (4)$$

How many mL of an ASA stock solution of 8.0 M are needed to prepare 4 mL of a diluted ASA solution of 0.5 M?

Answer key (Equations 5 and 6):

$$\text{M}_1 \times \text{V}_1 = \text{M}_2 \times \text{V}_2 \quad (5)$$

$$\text{V}_1 = \frac{\text{M}_2 \times \text{V}_2}{\text{M}_1} = \frac{0.5\ \text{M} \times 4\ \text{mL}}{8.0\ \text{M}} = 0.25\ \text{mL} \ (6)$$

Laboratory Component # 2:

What is the working principle of UV-Vis absorption spectrophotometry? Give two examples of applications for this instrumentation technique.

Answer key:

UV-Vis absorption spectrophotometry facilitate the quantification of an analyte (e.g., ASA in aspirin) with the help of the Beer-Lambert Law and external calibration curves for the analyte of interest. The Beer-Lambert law (Equation 7) relates to the attenuation (I) of the intensity of the incident light (I_0), at a specific wavelength, to the properties of the analyte material of an unknown concentration (*c)*, through which the light is travelling.

$$A = \log_{10} (I_0/I) = \varepsilon\ c\ l \qquad (7)$$

where l refers to the optical path length of the cuvette hosting the analyte, A is the recorded absorbance value of the analyte at the corresponding wavelength maximum (λ_{max}), and ε is the molar absorptivity of the analyte. "UV-VIS absorption is widely used in a multitude of applied and fundamental science applications such as monitoring the progress of a reaction and verifying the purity and composition of pharmaceutical drugs (i.e., quality control and quality assurance – QC/QA)."[1-3]

Calculate the concentration, c, of an analyte ASA using the Beer-Lambert law. Consider an absorbance of 0.618, a molar absorptivity of 1.432 $mM^{-1}cm^{-1}$, and a path length of 1 cm.

Answer key (Equations 8 and 9):

$$A = \varepsilon \cdot c \cdot l \quad (8)$$

$$c = \frac{A}{\varepsilon \cdot l} = \frac{0.618}{(1.432\ mM^{-1}cm^{-1}) \cdot (1\ cm)} = 0.432\ mM \quad (9)$$

Calculate the concentration of an analyte (ASA) using the calibration curve in Figure S1 and an absorbance value of 0.316.

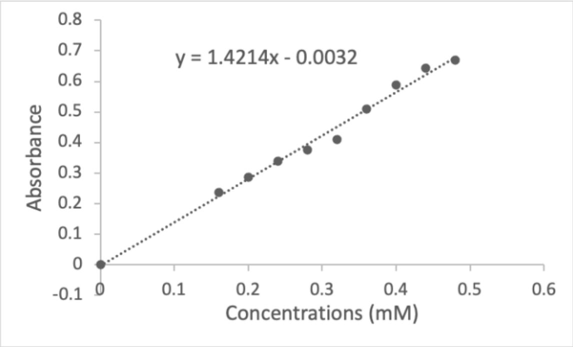

Figure S1. An illustrative external calibration curve constructed by a student with nine standards of ASA (0.000-0.480 mM) and one blank (0 mM of ASA) using parallel dilutions. The equation of the best fit line (y = mx + b) was inserted.

Answer key (Equation 10):

$$c = \frac{y+0.0032}{1.4214} = \frac{0.316+0.0032}{1.4214} = 0.225\ M \quad (10)$$

<u>*Laboratory component #3:*</u>

Using the hypothetical reaction data (rates of reaction, k) provided in Table S7 for the hydrolysis reaction of ASA at different temperatures, estimate the activation energy (E_a).

Table S7. Fictitious, practice kinetics data for the temperature (T) and the ln of the rate of reactions (k) in the hydrolysis of aspirin (ASA).

**ln (k)**	**1/(T) in 1/K**
-10.7244681	0.003470415
-9.903487553	0.003331667
-8.111728083	0.003094538
-5.80914299	0.002872325

Answer Key:

First, the rates of reaction values from Table S7 (ln k_{buffer} on the Y axis) were plotted in Figure S2 as a function of the reciprocal of the temperature (1/T on the X axis).

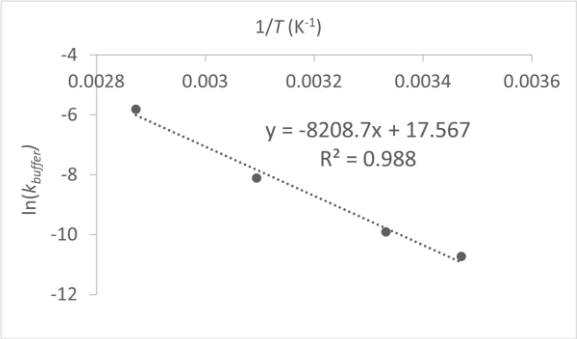

Figure S2. Graph showing the change in ln (k_{buffer}) as a function of 1/T (in 1/K units).

The slope of the best fit line in Figure S2 ($-E_a/R$ – Arrhenius Equation 11) was then multiplied by the universal gas constant, R of 8.314 J / (mol K), value to estimate the activation energy (E_a) as shown below (Equation 12).

$$\ln k_{buffer} = \ln A + \left(-\frac{Ea}{R}\right) \cdot \frac{1}{T} \qquad (11)$$

$$Ea = -(\text{slope} \times R) = -\left(-8208.7\ K\right) \times 8.314\ \frac{J}{mol \cdot K}\right) = \left(68247.13\ \frac{J}{mol}\right) \times \left(\frac{1\ kJ}{1000J}\right)$$

$$\cong 68.25\ \frac{kJ}{mol} \quad (12)$$

Explain why a pseudo-first order reaction is used to study a secondary order reaction kinetics, include experimental design differences?

Answer Key:

To reduce the complexity of second order reactions, one or more of the reactant components can be held at constant concentration. This component will result into a simplification of the related kinetics calculations by reducing the order of the reaction from second order to pseudo -first order.

2. During laboratory assessments

Tables S8-S12 represent the assessment tools, which were developed and utilized by the instructor for the evaluation of laboratory skills (LS1-LS5) during laboratory. Rating of each skill and its subcategories was on a Likert scale from 1 to 5 based on the students' level of competency in the observed lab skill. Instructors agreed that a rating of 5 corresponded to a high level of competence or expert proficiency (the student had extensive experience in the skill area), 4 to a moderately

high level of competence or advanced proficiency (the student had good experience in the skill area), 3 to an average level of competence or intermediate proficiency (the student had some experience in the skill area), 2 to a low level of competence or basic proficiency (the student had little experience in the skill area), and 1 to no level of competence or awareness (the student had no experience in the skill area). The arithmetic average class values were given for illustrative purposes for two different cohorts of chemistry students (n = 14 and n = 12 students). Because mean values (arithmetic averages – Table S8-S12) are more sensitive to outliers that can skew the average significantly, median values (positional averages - Table S13) were also estimated together with their corresponding modes. The mode is the most commonly occurring data point in a dataset, which contains numerous repetitive values such as the LS1-LS5 ratings. Percentile scores for values below the mean (Table S13) were also determined according to equation (13), where n is the number of values below the mean and N denotes the total number of values. The evaluation results were calibrated for each of the five basic and in-depth skills (LS1-LS5) by using the assessment criteria described in Tables S8-S12. Additionally, descriptive notes were taken by all instructors with respect to each of the five laboratory skills to facilitate the calibration process of the associated Likert scores. The Likert scale simply determined if the students had competency in a particular technique.

$$Percentile\ (\%) = (n/N) \times 100 \qquad (13)$$

The corona virus 19 (COVID-19) pandemic forced most higher education institutions to shift to remote or hybrid learning in both lecture and laboratory courses, in 2020-2021. As a result, the hands-on training in core science undergraduate laboratory courses was drastically diminished at our institution. The first cohort of chemistry students (Fall 2022), who completed this laboratory module, were found to be impacted to a larger degree by the COVID-19 pandemic than the second cohort of chemistry students (Fall 2024). This first cohort of students noted in their COVID-19-related surveys that virtual experiments were less valuable than traditional laboratory experiments to the overall chemistry learning experience. Upper-division undergraduate laboratory courses such as *Instrumental Analysis* and *Physical Chemistry*, where fine-tuned, hands-on skills or in person training on sizable/costly instruments are central to the experience, were the most impacted during the pandemic. The student surveys as well as related discussions with other science instructors helped identify the LS1-LS5 skills, which were targeted for improvement through the presented laboratory experiences. Thus, this laboratory module proved successful in addressing

these post-pandemic challenges with the upper-division, hands-on laboratory competency. Basic and advanced laboratory skills (LS1-LS5) were improved for both cohorts of students, but the most noticeable increases in students' performance during instructor demonstrations and subsequent laboratory practice were noticed for the first group of students, who were more impacted by the pandemic. Thus, additional practice with all basic techniques was provided to the first group of students, and the mean instructor ratings reflect this arrangement (see group #1 versus group #2 values in Tables S8-S11). Discrepancies were noticed in the rating values for the pre-wetting technique associated with LS3 – micropipetting (Table S10), which was practiced less with group #2 when compared to group #1. However, the second group of students performed very well in the operation of a modern, lab benchtop UV-Vis absorption spectrophotometer (LS5). During an informal class survey, we learned that this second cohort of students was better equipped with in-depth instrumentation skills. This hands-on experience was acquired post-pandemic through undergraduate research projects in research active laboratories.

Table S8. Mean instructor ratings and corresponding standard deviations (SD) of the observed laboratory skill LS1 – weighing among two different cohorts of students (*n* = 14 students in group #1 and n = 12 students in group #2).

**LS1 - Weighting**	**Group #1 Mean ± SD**	**Group#2 Mean ± SD**
Balance operation	4.57 ±1.16	4.29 ± 0.81

Table S9. Mean instructor ratings and corresponding standard deviations (SD) of the observed laboratory skill LS2 –solution handling among two different cohorts of students (*n* = 14 students in group #1 and n = 12 students in group #2).

**LS2 -Solution handling**	**Group #1 Mean ± SD**	**Group #2 Mean ± SD**
Storing	4.60 ± 1.20	4.29 ± 0.81
Following Instructions	4.60 ± 1.50	4.46 ± 0.88
Volume Measurement	4.20 ± 1.30	4.21 ± 1.02

Table S10. Mean instructor ratings and corresponding standard deviations (SD) of the observed laboratory skill LS3 – micropipetting among two different cohorts of students (*n* = 14 students in group #1 and n = 12 students in group #2).

**LS3 - Micropipetting**	**Group #1)**<br>**Mean ± SD**	**Group #2**<br>**Mean ± SD**
Contamination	4.40 ±0.90	4.21 ± 0.78
Pipette Angle	4.90 ± 0.40	3.79 ± 1.35
Storing Pipettes	4.10 ±1.50	4.58 ± 0.68
Pre-Wetting	4.00±1.52	2.56 ± 1.18
Change Tips	4.60 ±1.10	5.00 ± 0.00

Table S11. Mean instructor ratings and corresponding standard deviations (SD) of the observed laboratory skill LS4 - operation of a lab bench pH meter among two different cohorts of students (*n* = 14 students in group #1 and n = 12 students in group #2).

**LS4 – Operation of a pH meter**	**Group #1**<br>**Mean ± SD**	**Group #2**<br>**Mean ± SD**
Contamination	4.50±1.46	4.29 ± 0.95
Storage	3.93±1.49	4.83 ± 0.38

Table S12. Mean instructor ratings and corresponding standard deviations (SD) of the observed laboratory skill LS5 – operation of a modern, lab benchtop UV-Vis absorption spectrophotometer among two different cohorts of students (*n* = 14 students in group #1 and n = 6 students in group #2).

**LS5 – operation of a modern**<br>**UV-Vis absorption spectrophotometer**	**Group #1**<br>**Mean ± SD**	**Group #2**<br>**Mean ± SD**
Setup	3.30 ± 1.60	4.30 ± 0.82
Contamination	3.70 ± 2.00	4.90 ± 0.32
Handling	3.20 ± 2.10	4.70 ± 0.67

Table S13. Median instructor values, corresponding modes, and percentiles values below the mean for each laboratory sub-skill associated with LS1-LS5 in group #2 (n = 12 students).

**Laboratory Skill (LS)**	**Median**	**Modes**	**Percentile (%)**
LS1 – Weighting			
• Balance operation	5.0	5	43.75
LS2 -Solution handling			
• Storing	4.0	5	32.35
• Following Instructions	4.0	5	56.76
• Volume Measurement	4.50	5	50.00
LS3 – Micropipetting			
• Contamination	4.0	4	63.64
• Pipette Angle	4.0	5	44.12
• Storing Pipettes	4.5	5	50.00
• Pre-Wetting	2.0	2	64.71
• Change Tips	5.0	5	11.76
LS4 – Operation of a pH meter			
• Contamination	5.0	5	45.83
• Storage	5.0	5	16.67
LS5 – operation of a modern UV-Vis absorption spectrophotometer			
• Setup	4.0	4 & 5	62.50
• Contamination	5.0	5	31.25
• Handling	5.0	5	46.88

3. Post-laboratory assessments

All post-laboratory reports were successfully completed and graded by both the instructor and teaching assistants. The lab report questions, representative data collection tables, and answer keys for each laboratory component are provided below with a suggested grading rubric guide for a

total of 100 points. These illustrative examples were selected from the class responses. The average class score corresponded to a B letter grade (86-87%).

Points breakdown for the post-lab report (100 points total)

Laboratory Component #1 (15 points)

- Three data collection tables and related calculations (5 points each, Tables S14-S16)

Laboratory Component #1 (30 points)

- ASA concentration determinations from Beer-Lambert Law (15 points)
- ASA concentration determinations from the external calibration curve (15 points)

Laboratory Component #1 (55 points)

- Determination of the rate constant (*k*) for the pseudo-first order reaction (30 points)
- Determination of the activation energy (E_a) for the pseudo-first order reaction (25 points)

Laboratory Component #1 (15 points):

Representative student data are given below in Tables S14-S16 (*5 points for each of the data collection tables and related calculations)*.

Table S14. Representative data for the preparation of over-the-counter aspirin tablet solutions.

**Aspirin DG health (tablet brand) and 325 m of ASA**	**Whole aspirin tablet**	**1st Half tablet**	**2nd Half tablet**
Mass (g) of aspirin tablets	0.3699	0.1373	0.2294
pH of aspirin solution before $Fe(NO_3)_3$•$9H_2O$ addition		12.277	12.156
Mass of $Fe(NO_3)_3$•$9H_2O$ (g)		0.0477	0.0477
pH of aspirin solution after $Fe(NO_3)_3$•$9H_2O$ addition		1.638	1.643
Volume of HCl added (µL)		400	420
Volume of NaOH added (µL)		-	-

Table S15. Representative data for the preparation of ASA standards and blank using parallel dilutions.

Preparation of standards	Blank	Standard #1	Standard #2
pH of aspirin solution before $Fe(NO_3)_3$•$9H_2O$ addition	7.060	11.112	11.037
Mass of $Fe(NO_3)_3$•$9H_2O$ (g)	0.0480	0.0481	0.0475
pH of aspirin solution after $Fe(NO_3)_3$•$9H_2O$ addition	2.423	2.460	2.473
Volume of HCl added (µL)	420	430	440
pH after HCl addition	1.625	1.649	1.644

Table S16. Representative data for the preparation of ASA standards and blank using serial dilutions.

Molarity of standard (mM)	Volume of standard (mL)	Volume of DI water (mL)	Volume of HCl (µL)
0.480	2.40	50.00 – 2.40 = 47.6	200
0.440	45.83	50.00 - 45.83 = 4.17	200
0.400	45.45	50.00 – 45.45 = 4.55	250
0.360	45.00	50.00 – 45.00 = 5.00	250
0.320	44.40	50.00 – 44.40 = 5.60	250
0.280	43.75	50.00 – 43.75 = 6.25	300
0.240	42.86	50.00 – 42.86 = 7.14	350
0.200	41.60	50.00 – 41.60 = 8.40	400
0.160	40.00	50.00 – 40.00 = 10.00	450

Laboratory Component # 2 (30 points):

ASA concentration determinations from Beer-Lambert Law (15 points)

Students estimated the concentration of ASA (c) using the Beer-Lambert law (Equation 14) and the absorption spectrum of the iron (III) salicylate complex resulting from the hydrolysis of ASA (Figure S3). It should be noted that ASA hydrolysis to SA, which then binds to Fe^{3+} to form the iron (III) salicylate complex that is spectrophotometrically active in the visible (Vis) region.

$$A = \varepsilon \cdot c \cdot l \quad (14)$$

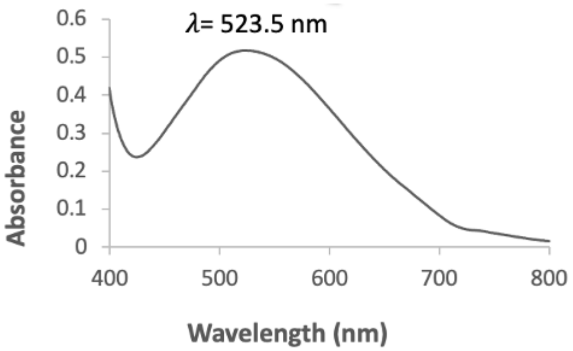

Figure S3. The absorption spectrum of the iron (III) salicylate complex (0.320 mM of ASA standard) showing a wavelength maximum, λ_{max}, at 523.5 nm and an absorbance value, A, of 0.519 at this maximum.

The absorbance (A = 0.519) was obtained from the scanned spectrum of ASA sample (Figure S3) at the maximum wavelength (λ_{max} = 523.5 nm), and the optical path length was (l = 1 cm). This was determined using the *Peak Label* function of the *Scan* application of the Cary 60 software and was later confirmed while plotting the spectrum file in Excel. The molar absorptivity coefficient (ε = 1.381 mM^{-1} cm^{-1}) was provided to students. Solving Equation 15 for c resulted into an ASA concentration of 0.376 mM. This corresponds to a percent difference of ~15.7%, when compared to the 0.325 mM concentration of ASA standard that was utilized in this illustrative, student example.

$$c = \frac{A}{\varepsilon \cdot l} = \frac{0.519}{(1.381\ \mathrm{mM^{-1}\ cm^{-1}}) \cdot (1\mathrm{cm})} = 0.376\ \mathrm{mM} \qquad (15)$$

ASA concentration determinations from the external calibration curve (15 points)

Figure S4 displays an illustrative external calibration curve, which was constructed using nine standards (0.160, 0.200, 0.240, 0.280, 0.320, 0.360, 0.400, 0.440, and 0.480 mM of ASA) and one blank (0 mM of ASA). The ε value was estimated from slope (*m*) of the external calibration curve of the ASA standards (y = mx + b).

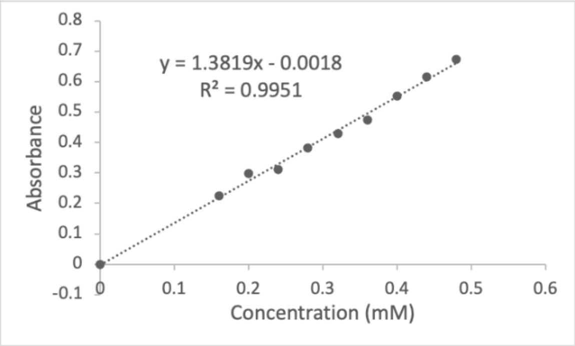

Figures S4. An illustrative external calibration curve constructed by students with nine calibration standards (0.000-0.480 mM of ASA) and one blank (0 mM of ASA) using parallel dilutions.

The ASA concentrations of the aspirin tablets were then determined through interpolation from the external calibration curve. Students were requested to complete these estimations both using Excel functions and a pocket calculator for further practice. An example is provided below (Equation 16) for the manual calculation of the ASA concentration (c) for the DG Health brand of aspirin. This corresponds to a percent difference of ~1.5% when compared to the amount of active ingredient listed on the DG Health label (325 mg of ASA).

$$c = \frac{y+0.0018}{1.3819} = \frac{0.4408+0.0018}{1.3819} = 0.320\text{ M} \qquad (16)$$

An illustrative absorption curve for the DG Health brand of aspirin is given below in Figure S5.

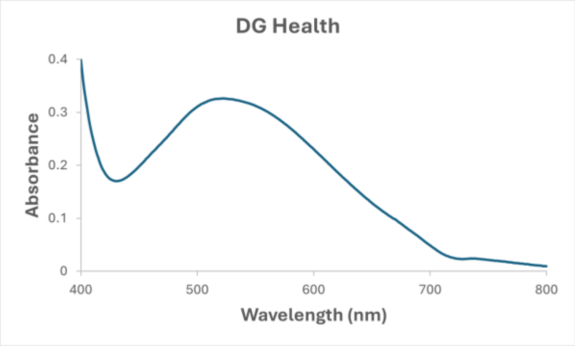

Figure S5. Illustrative absorption spectrum of the DG health aspirin tablet after the ASA hydrolysis to SA and subsequent binding of SA to Fe^{3+} to form the iron (III) salicylate complex.

<u>Laboratory Component # 3 (55 points):</u>

Determination of the rate constant (*k*) for the pseudo-first order reaction (30 points)

Figure S6 shows a representative absorption spectrum of SA (0.556 mM standard) having a wavelength maximum, λ_{max}, at ~293-295 nm and a corresponding absorbance value, A, of 2.7 at this maximum.

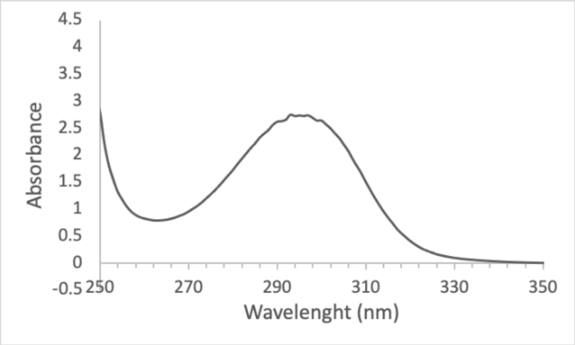

Figure S6. The absorption spectrum of 0.556 mM of SA standard showing a characteristic absorption maximum in the UV portion of the electromagnetic spectrum at ~293-295 nm.

An external calibration curve (Figure S7) was developed within the 0.0-1.0 mM range to facilitate the interpolation of the analyte SA concentrations (0.0-0.74 mM) of the aliquots collected during the kinetic experiments.

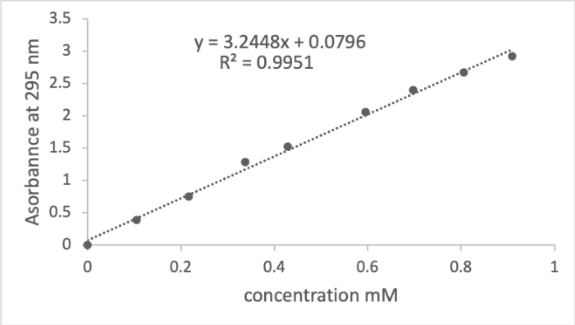

Figure S7. An illustrative external calibration curve constructed by students with the help of nine calibration standards (0.0 – 1.0 mM of SA) and one blank (0 mM of SA) through parallel dilutions. The equation of the best fit line (y = mx + b) and the corresponding R^2 value were inserted.

In this experiment, it is easier to measure the increasing concentration of the product, SA, than it is to measure the decreasing concentration of reactant, ASA. Therefore, this kinetics experiment followed the appearance of SA and related the SA concentration to the ASA concentration. One mole SA is produced when one mole of ASA degrades. Thus, aliquots of SA were collected at different time points and the absorbance (A) of the SA aliquots was measured at the same wavelength maximum of the external calibration curve (λ_{max} = ~293-295 nm). The absorbance

values were then plugged into the SA calibration curve equation to obtain the SA concentration at a specific temperature and each time t during the reaction. In the SA calculations, students were encouraged to use mass and molality rather than direct volume and molarity to eliminate temperature-dependent variations of the sample volume at 25, 50, 70, and 85°C. This indirectly monitors the concentration of ASA consumed over time (t). The remaining mass of ASA at time t was calculated by simply subtracting the SA concentration at time t from the initial amount of ASA at time 0 s and assuming an equimolar ratio of ASA and SA. The natural logarithm of remaining [ASA] versus time was then plotted (Figure S8). This resulted in a straight-line (Equation 17) with a negative slope, which is equal to the pseudo-first order rate constant (k).

$$y = -0.0003x - 7.1693 \qquad (17)$$

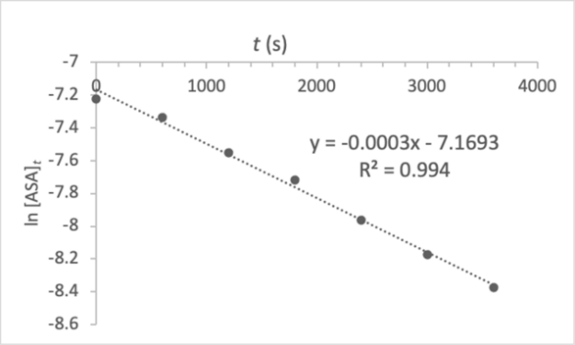

Figure S8. Student plot showing the changes in ln $[ASA]_t$ as a function of reaction time t (s) over a 60-min time interval at 75°C. Aliquot samples were measured every 10 min (n = 7 points).

Determination of the activation energy (E_a) for the pseudo-first order reaction (25 points)

The slope of the Arrhenius plot ($-E_a/R$, Figure S9) was utilized to calculate the activation energy, E_a, as described within the manuscript (Equations 18 and 19).

$$y = -8090.7x + 14.834 \qquad (18)$$

$$\text{Ea} = -(\text{slope} \times \text{R}) = -\left(-8090.7\ \text{K}) \times 8.314\ \frac{\text{J}}{\text{mol} \cdot \text{K}}\right) = \left(67266.07\ \frac{\text{J}}{\text{mol}}\right) \times \left(\frac{1\ \text{kJ}}{1000\text{J}}\right)$$

$$\cong 67.27\ \frac{\text{kJ}}{\text{mol}} \quad (19)$$

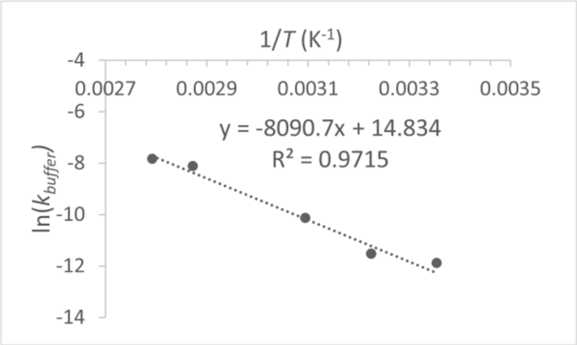

Figures S9. Student Arrhenius plot: natural logarithm of the measured rate constants, ln(k_{buffer}), versus the reciprocal of temperature, 1/T (K^{-1}), at 25, 37, 50, 75, and 85°C (n = 5 points).

4. Tentative timeline and additional notes for instructors

A tentative timeline (Table S17) is provided below for the three-laboratory components #1-3 of three (3) hours each. While the laboratory component #3 solely pertains to the Physical Chemistry curriculum, the laboratory components #1 and 2 target the curriculum requirements for hands-on practice in Analytical Chemistry. Laboratory components #1 and 2 were designed to build up and refine both laboratory skills and knowledge that were deemed essential to the successful completion of laboratory component #3. These laboratory components are flexible; thereby, they can be tailored to one or multiple lab work sessions of 2-3 hours each.

Table S17. Tentative time schedule for the three laboratory components of three (3) hours each. TA denotes the teaching assistant, who can help complete assigned laboratory activities to increase flexibility and allocated time for other student activities.

**Lab Component**	**Laboratory Activities**	**Time Period**
#1: *Preparation of samples and standards*	Preparation of a 10 mM of ASA stock solution<br>Preparation of over-the-counter aspirin tablet solutions<br>Preparation of ASA standards and blank using parallel dilutions and/or<br>Preparation of ASA standards and blank using parallel dilutions	15 min (TA)<br>1 hour<br>1 hour<br>45 min (TA)

***#2:** Concentration determinations by UV-Vis absorption spectrophotometry*	ASA concentration determinations from Beer-Lambert Law<br>ASA concentration determinations from the external calibration curve	45 min<br>2 hours<br>15 min
#3: Kinetics of aspirin hydrolysis	Preparation of a 10x PBS (pH of 7.4) at room temperature<br><br>Preparation of blank and SA standards using parallel dilutions or<br>Preparation of blank and SA standards using serial dilutions<br>SA spectrum scan<br>SA concentration determination from the external calibration curve<br>Hydrolysis reaction of aspirin (ASA) at different temperatures (25, 37, 50, 75, and 85°C) and constant pH (7.4±0.4)	15 min (TA)<br><br>45 min (TA)<br><br>15 min<br>30 min<br><br>1 hour 15 min

Additional notes for instructors

- The POGIL-based approach was successfully developed according to the five essential features laid by the National Research Council for inquiry-based learning and teaching in science and engineering courses. By this means, student learners *"(1) are engaged by scientifically oriented questions, (2) give priority to evidence, which allows them to develop and evaluate explanations that address scientifically oriented questions, (3) formulate explanations from evidence to address scientifically oriented questions, (4) evaluate their explanations in light of alternative explanations, particularly those reflecting scientific understanding, and (5) communicate and justify their proposed explanations."*[4]
- The iron (III) nitrate nonahydrate compound is strongly hygroscopic. Therefore, it is imperative to store it in a desiccator and to instruct students about its correct handling.
- Demonstrating first the laboratory skills (LS1-LS5) is key to the successful completion of the experiments within the allocated time. It will help remediate or refresh these skills.
- A standard operating procedure (SOP) was prepared beforehand and was shared with the students as pre-laboratory material. It was closely followed during the in-class measurements

to avoid instrument damage and for good laboratory practices. It was provided as supporting material for instructors and editorial office only.

- Both serial and parallel dilutions could be utilized for additional micropippeting practice. This practice could be tailored to the needs of the students and the available lab time. Serial dilutions can be generally completed faster but can also lead to errors if students are inexperienced. Parallel dilutions take normally longer but can help build up skills through additional practice. Unlike serial dilutions, parallel dilutions permit the independent correction of standard outliers on the external calibration curve. Additional lab time periods can be devoted to reviewing and improving the construction of a quality ($R^2 \geq 0.995$) calibration curve with a relevant number of standards (n = 9-11).
- The ASA standards and aspirin samples should be used fresh (one week after their preparation). The ASA standards should be allowed to complex first to the Fe^+(III) ions (a few minutes as indicated by the change in color) before proceeding with the UV-Vis absorption spectrophotometer measurements.
- A single over-the-counter aspirin tablet was assigned to each laboratory group (2-3 students) to ensure the timely completion of the assigned tasks. Data collected on various aspirin brands were then exchanged among students. Over-the-counter aspirin tablets should be labelled as "For Research Purpose Only, Not for Human Consumption".
- A Cary 60 UV-Vis absorption spectrophotometer (Agilent Inc., at UV-Vis Spectrophotometer, Routine UV-Vis, Cary 60 | Agilent) was utilized for the concentration determinations and the kinetics measurements in this laboratory module. Alternative devices that could be used include but are not limited to the Genesys 40/50 Vis/UV-Vis spectrophotometer (Thermo Fisher Scientific, at Thermo Scientific GENESYS 40/50 Vis/UV-Vis Spectrophotometers:Spectroscopy:Spectrophotometry | Fisher Scientific) and the UV-Vis spectrometer SE-3607 for science education (Pasco, at UV-Vis Spectrometer - SE-3607 - Products | PASCO).
- It is recommended that students should evaluate both the molarity and molality of the heated solutions.
- Laboratory component #3 can be implemented for a pH-dependent study.

References

[1] Drago, R. S. *Physical Methods for Chemists*, 2nd Ed.; Saunders College Publishing: Orlando, Florida, U.S.A., 1992.

[2] Gandhimathi, R.; Vijayaraj, S.; Jyothirmaie, M. P. Analytical Process of Drugs by Ultraviolet (UV) Spectroscopy – A Review. *IJPRA* **2012**, *2* (2), 72-78.

[3] Skoog, D. A.; Holler, James F.; Crouch, St. R. *Principles of Instrumental Analysis*, 7th Ed.; Cengage Learning: Boston, 2017; pp. 304-331.

[4] Olson, S., Loucks-Horsley, S., Eds.; *Inquiry and the National Science Education Standards: A Guide for Teaching and Learning*; National Academy Press: Washington, DC, 2000.
